# No evidence that averaging voices influences attractiveness

**DOI:** 10.1038/s41598-024-61064-9

**Published:** 2024-05-07

**Authors:** Jessica Ostrega, Victor Shiramizu, Anthony J. Lee, Benedict C. Jones, David R. Feinberg

**Affiliations:** 1 Psychology, Neuroscience and Behaviour, McMaster University, Hamilton, Canada; 2 Department of Psychological Sciences and Health, University of Strathclyde, Glasgow, UK; 3 Division of Psychology, University of Stirling, Stirling, UK

**Keywords:** Psychology, Human behaviour

## Abstract

Vocal attractiveness influences important social outcomes. While most research on the acoustic parameters that influence vocal attractiveness has focused on the possible roles of sexually dimorphic characteristics of voices, such as fundamental frequency (i.e., pitch) and formant frequencies (i.e., a correlate of body size), other work has reported that increasing vocal averageness increases attractiveness. Here we investigated the roles these three characteristics play in judgments of the attractiveness of male and female voices. In Study 1, we found that increasing vocal averageness significantly decreased distinctiveness ratings, demonstrating that participants could detect manipulations of vocal averageness in this stimulus set and using this testing paradigm. However, in Study 2, we found no evidence that increasing averageness significantly increased attractiveness ratings of voices. In Study 3, we found that fundamental frequency was negatively correlated with male vocal attractiveness and positively correlated with female vocal attractiveness. By contrast with these results for fundamental frequency, vocal attractiveness and formant frequencies were not significantly correlated. Collectively, our results suggest that averageness may not necessarily significantly increase attractiveness judgments of voices and are consistent with previous work reporting significant associations between attractiveness and voice pitch.

## Introduction

Vocal attractiveness influences a diverse range of important social outcomes. For example, people with more attractive voices are perceived to be more effective leaders^1,2^, favored in hiring decisions^3,4^, and preferred as romantic partners^5–9^. Consequently, many researchers have attempted to identify acoustic characteristics that influence judgments of vocal attractiveness (reviewed in Pisanski and Bryant)^6^.

Most research that has attempted to identify acoustic characteristics that influence vocal attractiveness has focused on the possible roles played by fundamental frequency (i.e., pitch) and formant frequencies (i.e., a correlate of body size). Both characteristics are sexually dimorphic, with male voices possessing (on average) lower fundamental frequency and formant frequencies than female voices^10–13^. Both correlational and experimental studies have reported that more attractive male voices tend to have lower fundamental frequencies^12,14–17^, and that more attractive female voices tend to have higher fundamental frequencies^5,14,15,18–21^. Although some studies have reported preferences for male voices with more masculine formant frequencies^21,22^ and female voices with more feminine formant frequencies^21,23^, other studies did not replicate these patterns of results^12,24^.

While the studies described above investigated the roles sexually dimorphic acoustic characteristics might play in vocal attractiveness judgments, other work has tested for possible effects of vocal averageness. Bruckert et al.^25^ reported that increasing averageness of voices (i.e., making them more prototypical) increased the attractiveness of both male and female voices. This positive effect of averageness on judgments of vocal attractiveness is similar to the positive effects of averageness that have been widely reported in the facial attractiveness literature and are thought to occur because of the greater fluency with which average stimuli can be processed^26,27^. However, there has not yet been a published replication of this effect of averageness on vocal attractiveness judgments.

In light of the above, we manipulated averageness in recordings of male and female voices using the same methods employed by Bruckert et al.^25^. First, in Study 1, we tested whether increasing vocal averageness caused voices to be perceived as less distinctive, as has previously been reported in studies using face images as stimuli^28–33^. We carried out this study to establish whether the listeners could perceive the averageness manipulation. Next, in Study 2 and using the same stimuli we used in Study 1, we tested whether increasing vocal averageness caused voices to be perceived as more attractive, as was previously reported^25^. Finally, in Study 3, we tested for possible relationships between the attractiveness ratings collected in Study 2 and fundamental frequency. In Study 3, we also tested for possible relationships between attractiveness ratings and estimates of vocal tract length derived from measured formant frequencies.

## Study 1

Previous research has found that increasing facial averageness decreases distinctiveness ratings of faces^28–33^. Consequently, in Study 1, we tested whether increasing vocal averageness has a negative effect on distinctiveness ratings of voices. This result would demonstrate that our manipulation of vocal averageness could be detected by listeners and influences their perceptions of the voices.

### Methods

Protocols for the 3 studies were approved by Department of Psychological Sciences and Health (University of Strathclyde) Ethics Committee (51/21/05/2021/A), and the McMaster University Research Ethics Board (2008-107/6248). All participants provided informed consent before participation in each study. All data, analysis code, and the full outputs for all analyses for each study are publicly available on the Open Science Framework (https://osf.io/pxc82/).

#### Voice stimuli

**Figure 1:**
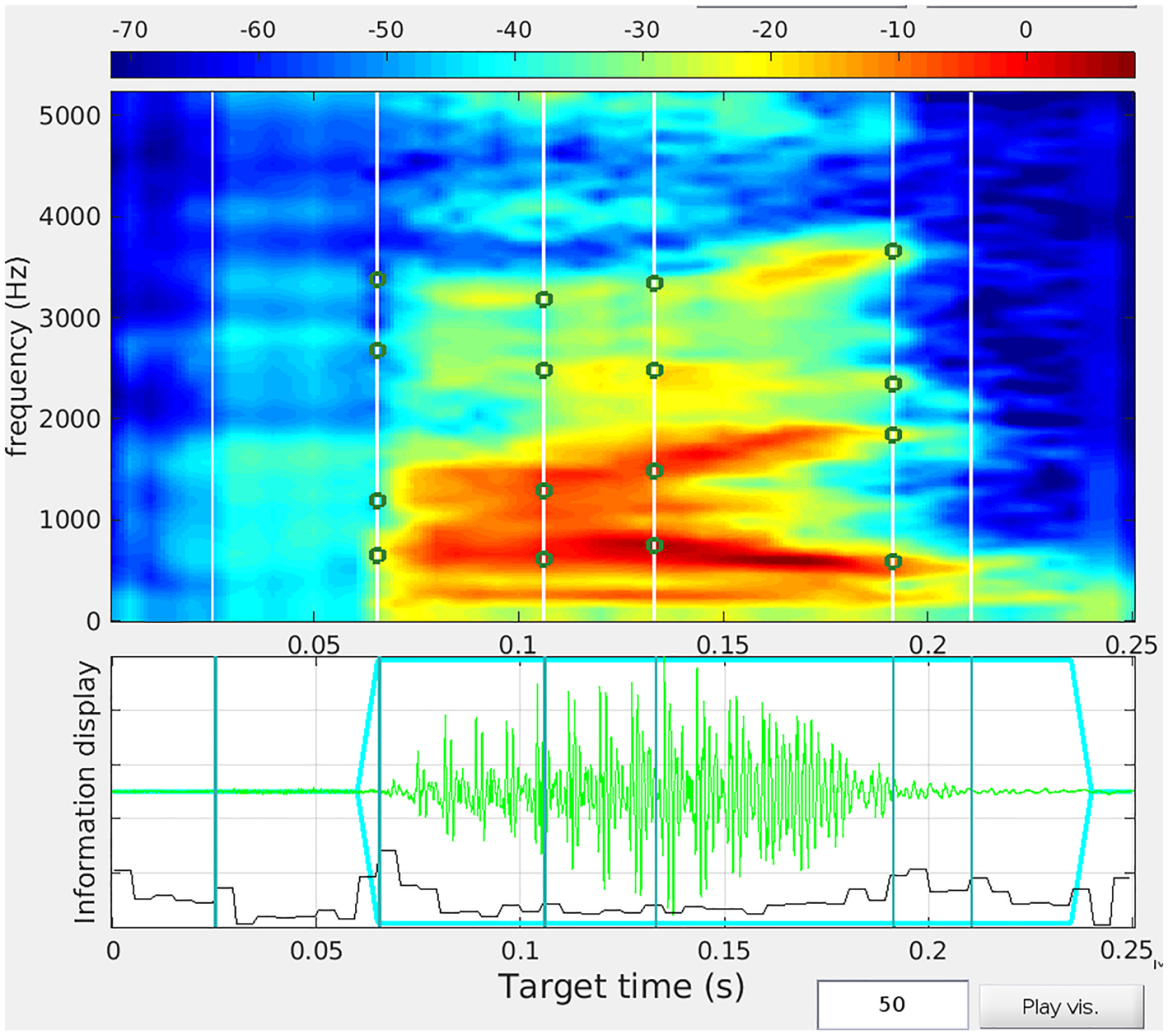
Part of the morphing procedure is to demarkate key parts of the time and frequency space in the spectrogram (x is time, y is frequency, z is amplitude). On the top panel, the white vertical lines demarkate time points. The first is the onset of the “H” sound, the second line is the offset of the “H” and the onset of the “I”. I is a dipthong vowel, and we mark the formant frequency transition in frequency using the dots, and in time using the lines. The final line represents the end of the word, “Hi”. The bottom panel represents the time points on an oscillogram (x is time, y is power). This demarkation procedure was done to each file individually and manually checked for accuracy.

We recorded 32 male and 32 female students at McMaster University saying “Hi” using a Sennheiser MKH-800 condenser microphone with phantom power and cardioid pickup pattern in a whisper-room sound booth. We recorded sounds at 96 kHz sampling rate at 24-bit amplitude quantization. Following Bruckert et al.^25^, we then used n-way morphing in Tandem-STRAIGHT^33^ to morph voices to manipulate vocal averageness. The software first decomposes the sound into pitch, duration, aperiodicity (e.g., the H in Hi), formant frequencies, and spectrum level. Each of these acoustic characteristics are then averaged separately. This procedure is analogous to the procedure used for face morphing, in that we visually represent the sound using a spectrogram (time is on the X axis, frequency on the Y axis, and amplitude is represented by colour or shading). We demarkated and aligned the parts of sound in time so that we only average the “H” portion of “Hi” with other “H”’s, and we only average “I”’s with other “I’s. We also demarcate frequency space to make sure we average the pitch with the pitch, each formant (1–4) with its corresponding formant in each file (see Fig. 1).

From the 32 male voices, we created 16 averages of 2 male voices, 8 averages of 4 male voices, 4 averages of 8 male voices, 2 averages of 16 male voices, and 1 average of 32 male voices. From the 32 female voices, we created 16 averages of 2 female voices, 8 averages of 4 female voices, 4 averages of 8 female voices, 2 averages of 16 female voices, and 1 average of 32 female voices. Averages were synthesized at 44.1 kHz sampling rate and 16-bit amplitude quantization. Finally, all 126 voices (i.e., both the averages and the original individual voices from which the averages were manufactured) were normalized to 70 dB RMS using VoiceLab^34^.

#### Distinctiveness ratings

Forty-nine male participants (Mean age = 26.45 years, SD = 8.54 years) and 47 female participants (Mean age = 25.96 years, SD = 7.98 years) rated all 126 voices (63 male voices and 63 female voices) for distinctiveness using a 7-point scale on which higher scores corresponded to higher distinctiveness. Following prior work^25^, male and female voices were presented in separate blocks of trials. Both trial order and block order were fully randomized. Participants were required to play a voice in full before they could rate it and were free to play each voice as many times as they wanted to before rating it. The study was run online, with participants recruited via Prolific. All participants reported having English as their first language. Inter-rater agreement was high for both male and female voices (both Cronbach’s alphas > 0.90).

### Results

**Figure 2:**
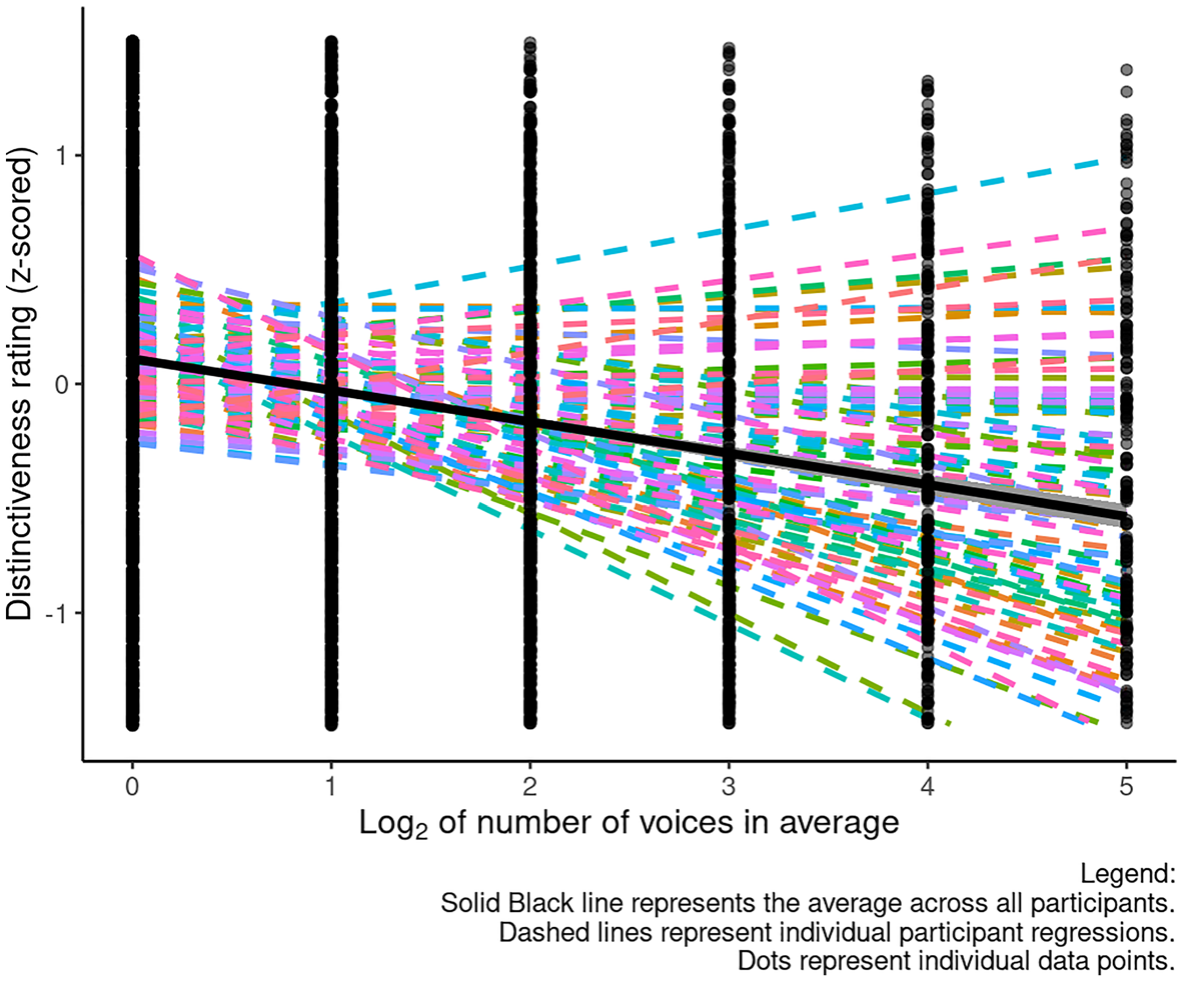
The significant negative effect of averageness level on distinctiveness ratings of voices (Study 1). The gray shading around the line represents the 95% confidence interval. Dashed lines represent individual regressions for each participant. The solid line represents the average for the group.

Analyses were conducted using R^35^ and the packages tidyverse 1.3.1^36^ , kableExtra 1.3.4^37^, lmerTest 3.1-3^38^ , jtools 2.2.3^39^, and stringr 1.5.0^40^. All data, analysis code, and the full outputs for all analyses are publicly available on the Open Science Framework (https://osf.io/pxc82/). Following Bruckert et al.^25^, each participant’s ratings were first converted to z-scores. We then analyzed distinctiveness ratings using a linear mixed effects model in which voice gender (effect coded so that male corresponded to -0.5 and female corresponded to + 0.5), rater gender (effect coded so that male corresponded to -0.5 and female corresponded to + 0.5), log_2_ of the averageness level (i.e., log_2_ of the number of voices from which a given stimulus was manufactured; 1, 2, 4, 8, 16, or 32, resulting in an x axis with points: 0, 1, 2, 3, 4, 5), the interaction between voice gender and averageness level, the interaction between rater gender and log_2_ averageness level, and the interaction among voice gender, rater gender, and averageness level were included as predictors. The model also included by-rater and by-stimuli random intercepts, by-rater random slopes for the interaction between voice gender and averageness level, and by-stimuli random slopes for rater gender. As we have fewer data points as the number of voices in the average increases, as such voice categories with more exemplars have stronger effects on the analysis. The random effects structure that we used for this analysis was based on recommendations^41,42^. These results are summarized in Table 1. The significant negative effect of averageness level on distinctiveness ratings is shown in Fig. 2.

**Table 1:** Results of our analysis of voice averageness and distinctiveness ratings (Study 1).

	Estimate	SE	t	df	*p*
Intercept	0.171	0.027	6.328	127.628	< 0.001
Averageness	− 0.189	0.021	− 9.071	154.805	< 0.001
Voice gender	0.055	0.070	0.789	192.632	0.431
Rater gender	− 0.002	0.025	− 0.073	57.849	0.942
Averageness × voice gender	− 0.018	0.036	− 0.499	135.036	0.618
Averageness × rater gender	0.001	0.027	− 0.039	68.351	0.969
Rater gender × voice gender	− 0.014	0.102	− 0.139	105.982	0.890
Averageness × voice gender × rater gender	− 0.008	0.035	− 0.234	82.128	0.815

In the analyses described above, averageness level was coded to reflect log_2_ of the number of voices from which a given stimulus was manufactured. An alternative way to code averageness level would be to code it on the number of voices in the averages, with points at 1, 2, 4, 8, 16, and 32. Repeating our initial analysis with this alternative coding scheme also showed a significant negative effect of averageness. Results for this analysis are reported in full at https://osf.io/pxc82/.

## Study 2

Consistent with previous research showing that increasing facial averageness significantly decreases distinctiveness ratings of faces^28–32^, increasing voice averageness had a significant negative effect on distinctiveness ratings of voices in Study 1. This result confirms that listeners could detect our averageness manipulation and that it could influence their perceptions of the voices we used in the testing paradigm we employed sufficiently well to produce a significant effect of averageness. In Study 2, we tested whether increasing vocal averageness had a significant positive effect on attractiveness ratings, as has previously been reported by Bruckert et al.^25^.

### Methods

Stimuli, methods, and participant recruitment were identical to those used in Study 1, except that a different group of 49 male participants (Mean age = 27.06 years, SD = 4.28 years) and 50 female participants (Mean age = 27.44 years, SD = 4.68 years) rated the voices for attractiveness using a 7-point scale on which higher scores corresponded to higher attractiveness. Inter-rater agreement was high for both male and female voices (both Cronbach’s alphas > 0.87).

### Results

**Figure 3:**
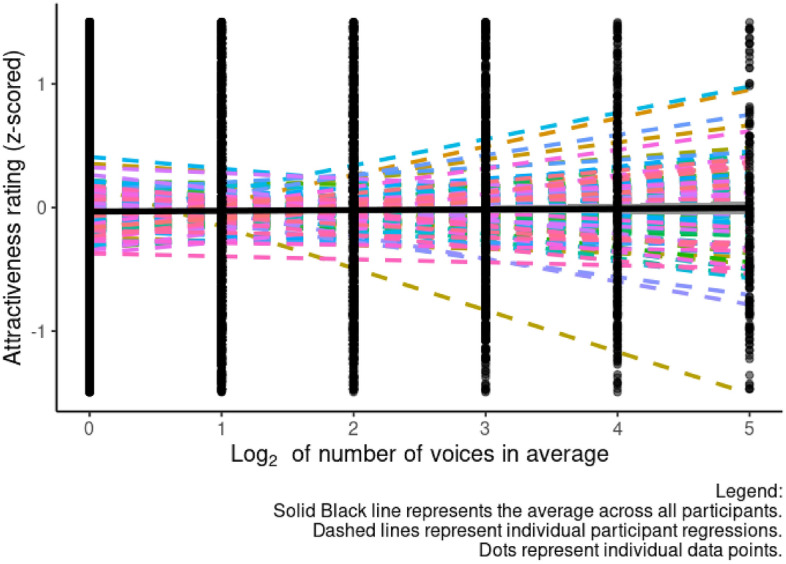
The non-significant effect of averageness level on attractiveness ratings (Study 2). The gray shading represents the 95% confidence interval. Dashed lines represent individual participants, solid lines represent the average across all participants.

Attractiveness ratings were analyzed in the same way as distinctiveness ratings were analyzed in Study 1. All data, analysis code, and the full outputs for all analyses are publicly available on the Open Science Framework (https://osf.io/pxc82/). Results of our initial analysis are summarized in Table 2. The non-significant effect of averageness level on attractiveness ratings is shown in Fig. 3. To interpret the significant interaction between averageness and rater gender in our initial analysis, we next ran separate analyses for male and female voices. Results of these analyses showed that the significant interaction between averageness and rater gender in our initial analysis reflected averageness having a nonsignificant positive effect on attractiveness ratings made by female raters (estimate = 0.023, SE = 0.027, t = 0.861, df = 136.639, *p* = 0.3391) and a nonsignificant negative effect on attractiveness ratings made by male raters (estimate = − 0.030, SE = 0.024, t = − 1.261, df = 117.882, *p* = 0.210).

**Table 2:** Results of our analysis of voice averageness and attractiveness ratings (Study 2).

	Estimate	SE	t	df	*p*
Intercept	0.003	0.031	0.108	125.654	0.914
Averageness	− 0.004	0.021	− 0.175	136.631	0.862
Voice gender	0.321	0.084	3.819	206.085	< 0.001
Rater gender	− 0.048	0.024	− 1.982	125.065	0.050
Averageness × voice gender	0.020	0.041	0.484	126.621	0.629
Averageness × rater gender	0.053	0.019	2.841	128.247	0.005
Rater gender × voice gender	− 0.629	0.125	− 5.033	115.231	< 0.001
Averageness × voice gender × rater gender	− 0.058	0.034	− 1.727	87.275	0.088

When we repeated our analyses with averageness level coded on the un-logged scale (1, 2, 4, 8, 16, 32). None of these models showed a significant effect of averageness. Results for these analyses are reported in full at https://osf.io/pxc82/.

## Study 3

Although we found that increasing averageness significantly decreased perceptions of voice distinctiveness in Study 1, we did not see a significant positive effect of averageness on vocal attractiveness in Study 2. This null result for averageness and attractiveness contrasts with Bruckert et al.^25^, who reported a significant positive effect of voice averageness on attractiveness ratings.

Bruckert et al.^25^found the most attractive pitch was 120 Hz, but most studies have found that male voices with lower-than-average pitch are more attractive. Indeed, previous studies have reported that fundamental frequency (i.e., voice pitch) is negatively correlated with male vocal attractiveness and positively correlated with female vocal attractiveness^6,14,17,19,22^ and/or that formant frequencies (an acoustic marker of vocal tract length) are negatively correlated with male vocal attractiveness and positively correlated with female vocal attractiveness^23–25^. Consequently, Study 3 tested for possible relationships between the vocal attractiveness ratings collected in Study 2 and both fundamental frequency (F0) and estimated vocal tract length (VTL) derived from measured formant frequencies.

### Methods

First, fundamental frequency (F0) was measured for each of the voices used as stimuli in Study 1 and Study 2. F0 was measured measured using Praat’s autocorrelation algorithm, accessed by VoiceLab software^36^. Next, an estimate of vocal tract length (VTL) was calculated for each of each of the voices used as stimuli in Study 1 and Study 2. To estimate VTL, formant frequencies were measured using Praat’s LPC Burg algorithm and converted to an estimate of VTL using a method described in Reby et al.^44^. There were significant sex differences in both F0 (t = 39.47, df = 124, *p* < 0.001) and estimated VTL (t = − 8.60 df = 124, *p* < 0.001), with F0 being higher in female voices (M = 229.74 Hz, SD = 16.29 Hz) than male voices (M = 115.27 Hz, SD = 16.27 Hz) and estimated VTL being lower in female voices (M = 16.74 cm, SD = 0.95 cm) than male voices (M = 18.18 cm, SD = 0.92 cm).

### Results

We tested for possible effects of fundamental frequency (F0) and estimated vocal tract length (VTL) on attractiveness ratings by analyzing the attractiveness ratings from Study 2 using linear mixed effects models with the same structure as those used to analyze distinctiveness and attractiveness ratings in our previous studies. We tested for possible effects of F0 and VTL in separate models. By contrast with the linear mixed effects models in Study 1 and Study 2, in Study 3 either F0 or VTL replaced averageness in our models. F0 and VTL were z scored within each sex prior to analyses. Two values for F0 and one value for VTL were more than three standard deviations from the respective means for the sample and were adjusted (i.e., winsorized) to be three standard deviations from the mean prior to analyses. Data, analysis code, and full results for all analyses are available on the Open Science Framework (https://osf.io/pxc82/).

**Figure 4:**
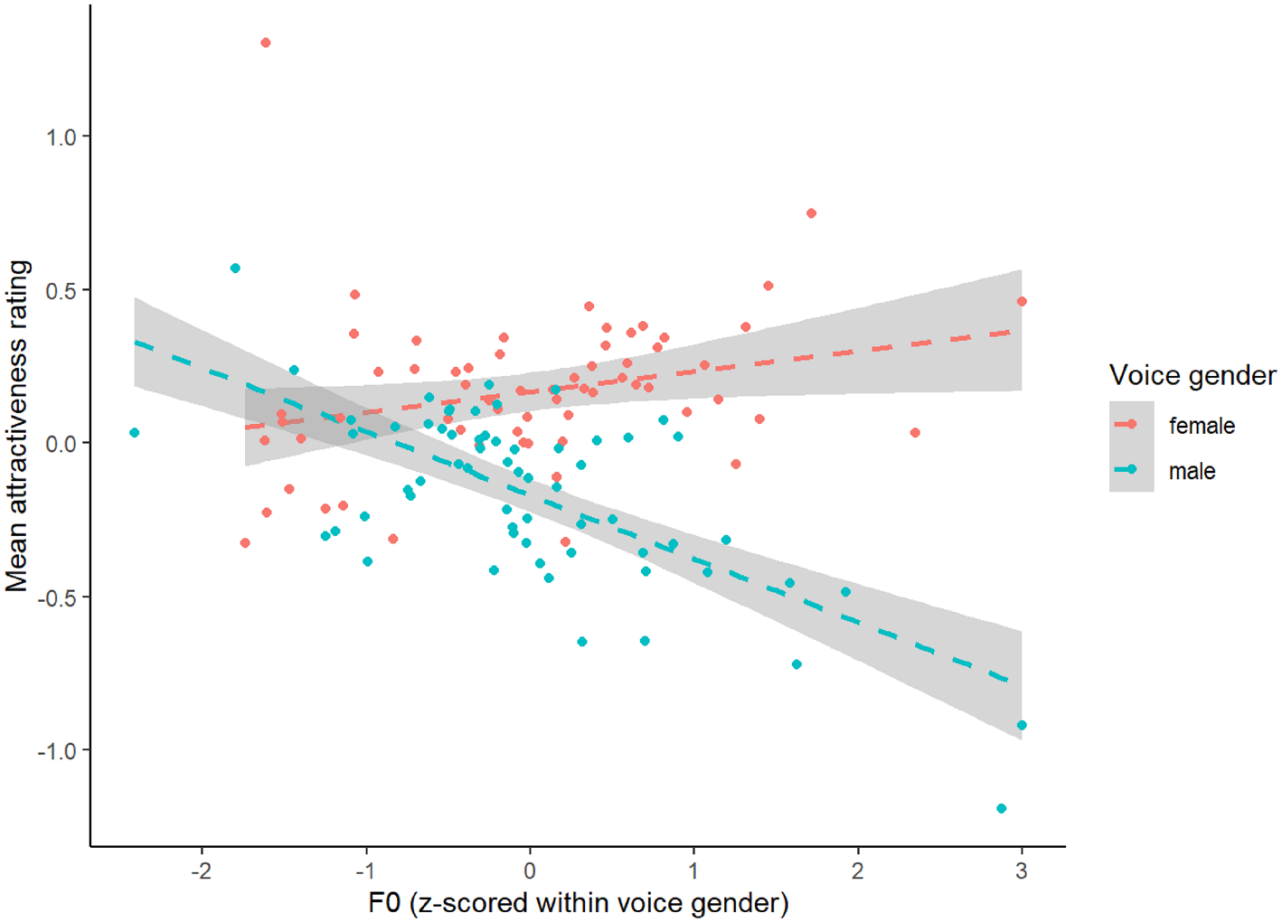
The significant relationships between fundamental frequency (F0) and attractiveness ratings of male and female voices in study 3. The gray shading represents the 95% confidence interval.

Results of our test for possible effects of F0 are shown in Table 3. To interpret the significant interaction between voice gender and F0 we repeated the analyses, this time analyzing data for male and female voices in separate models. These analyses indicated that the significant interaction between voice gender and F0 reflected F0 being positively and significantly correlated with women’s vocal attractiveness (estimate = 0.069, SE = 0.033, t = 2.085, df = 79.040, *p* = 0.040), but negatively and significantly correlated with men’s vocal attractiveness (estimate = − 0.211, SE = 0.033, t = − 6.335, df = 105.034, *p* < 0.001). These latter results are shown in Fig. 4. To interpret the significant interaction between rater gender and F0 we repeated the analyses, this time analyzing data for male and female raters in separate models. These analyses indicated that the significant interaction between rater gender and F0 reflected F0 being negatively and significantly correlated with vocal attractiveness ratings made by female raters (estimate = − 0.142, SE = 0.031, t = − 4.569, df = 113.201, *p* < 0.001), but not male raters (estimate = − 0.001, SE = 0.026, t = − 0.028, df = 110.944, *p* = 0.977). Here, including a term to account for whether voices were averaged or unmanipulated did not change the pattern of results (see supplementary material for results).

**Table 3:** Results of our analysis of fundamental frequency (F0) and attractiveness ratings (Study 3).

	Estimate	SE	t	df	*p*
Intercept	− 0.001	0.020	− 0.070	125.474	0.944
F0	− 0.071	0.024	− 2.94	173.15	0.004
Voice gender	0.342	0.071	4.843	164.581	< 0.001
Rater gender	− 0.001	0.016	− 0.071	123.863	0.944
F0 × voice gender	0.281	0.046	6.116	157.862	< 0.001
F0 × rater gender	− 0.141	0.030	− 4.752	95.612	< 0.001
Rater gender × voice gender	− 0.679	0.120	− 5.674	99.644	< 0.001
F0 × voice gender × rater gender	0.055	0.051	1.074	90.893	0.286

Results of our test for possible effects of estimated VTL are shown in Table 4. To interpret the significant interaction between rater gender and VTL we repeated the analyses, this time analyzing data for male and female raters in separate models. These analyses indicated that the significant interaction between rater gender and VTL reflected the nonsignificant positive effect of VTL on attractiveness ratings made by female raters (estimate = 0.012, SE = 0.031, t = 0.406, df = 129.852, *p* = 0.686) and the nonsignificant negative effect of VTL on attractiveness ratings made by male raters (estimate = − 0.036, SE = 0.025, t = − 1.448, df = 121.426, *p* = 0.150).

**Table 4:** Results of our analysis of estimated vocal tract length (VTL) and attractiveness ratings (Study 3).

	Estimate	SE	t	df	p
Intercept	− 0.000	0.024	− 0.000	125.654	1.000
VTL	− 0.012	0.025	− 0.463	137.598	0.644
Voice gender	0.339	0.075	4.493	185.829	< 0.001
Rater gender	0.000	0.020	0.001	125.115	1.000
VTL × voice gender	0.040	0.050	0.796	134.774	0.428
VTL × rater gender	0.048	0.024	2.038	104.193	0.044
Rater gender × voice gender	− 0.682	0.122	− 5.584	106.266	< 0.001
VTL × voice gender × rater gender	0.013	0.046	0.283	101.090	0.778

## Discussion

In Study 1 and Study 2 we tested for possible effects of vocal averageness on perceptions of distinctiveness (Study 1) and attractiveness (Study 2). We found that increasing vocal averageness significantly decreased distinctiveness ratings but did not have a significant effect on attractiveness ratings. That increasing averageness significantly decreased the perceived distinctiveness of voices is consistent with previous studies reporting negative effects of averageness on perceptions of the distinctiveness of faces^28–33^. The significant negative effect of averageness on distinctiveness ratings observed in Study 1 also demonstrates that listeners on average could perceive (i.e., detect) our manipulation of vocal averageness in this testing paradigm such that a significant effect could be obtained. Importantly, we note here that this significant effect does not necessarily imply that averaging would be detectable under more naturalistic (i.e., ‘real world’) listening conditions. Although we showed that listeners could detect our averageness manipulation in the testing paradigm we used, we did not replicate Bruckert et al.’s finding that increasing vocal averageness increased attractiveness ratings^25^. Thus, our results suggest that averaging does not necessarily significantly increase ratings of vocal attractiveness.

In Study 3, we tested for possible relationships between vocal attractiveness and both fundamental frequency (i.e., pitch) and formant frequencies. We first replicated the well-established findings that male voices tend to have significantly lower fundamental frequencies and significantly lower formant frequencies than female voices^10–13^. Consistent with previous work, we also found that more attractive male voices tended to have lower fundamental frequencies^12,14–17^ and that more attractive female voices tended to have higher fundamental frequencies^5,14,15,18–21^. That the negative effect of pitch on male vocal attractiveness was greater than the positive effect of pitch on female vocal attractiveness is also consistent with previous work^7^. By contrast with our results for fundamental frequency, formant frequencies did not predict either male or female vocal attractiveness in our study^12–24^. Thus, although we do not replicate a significant effect of averaging voices on attractiveness ratings, we do replicate the previously reported effect of voice pitch (i.e., it does not appear that there is something inherently unusual or atypical about this particular sample of raters or voices). Others have used voice cloning to create average voices using different speech types (phonemes, non-words, and sentences)^43^.

To summarize, although we found that increasing vocal averageness significantly decreased distinctiveness ratings (Study 1), we found no evidence that increasing vocal averageness significantly increased attractiveness ratings (Study 2). While these results show that participants could, on average, detect our averageness manipulation in the testing paradigm employed, the null results for attractiveness contrast with the finding that increasing vocal averageness increased attractiveness^25^. We also found that fundamental frequency (but not formant frequencies) significantly predicted vocal attractiveness, being negatively correlated with male vocal attractiveness and positively correlated with female vocal attractiveness (Study 3). Thus, while we found no evidence that averageness significantly increased vocal attractiveness, our results are consistent with the claim that pitch influences attractiveness judgments of voices.

## Data Availability

All data, analysis code, and the full outputs for all analyses for each study are publicly available on the Open Science Framework (https://osf.io/pxc82/).
